# Feeding and Dispersal Behavior of the Cotton Leafworm, *Alabama argillacea* (Hübner) (Lepidoptera: Noctuidae), on Bt and Non-Bt Cotton: Implications for Evolution and Resistance Management

**DOI:** 10.1371/journal.pone.0111588

**Published:** 2014-11-04

**Authors:** Francisco S. Ramalho, Jéssica K. S. Pachú, Aline C. S. Lira, José B. Malaquias, José C. Zanuncio, Francisco S. Fernandes

**Affiliations:** 1 Unidade de Controle Biológico, Embrapa Algodão, Campina Grande, Paraíba, Brazil; 2 Departamento de Entomologia, Universidade Federal de Viçosa, Minas Gerais, Brazil; Institute of Vegetables and Flowers, Chinese Academy of Agricultural Science, China

## Abstract

The host acceptance of neonate *Alabama argillacea* (Hübner) (Lepidoptera: Noctuidae) larvae to Bt cotton plants exerts a strong influence on the potential risk that this pest will develop resistance to Bt cotton. This will also determine the efficiency of management strategies to prevent its resistance such as the “refuge-in-the-bag” strategy. In this study, we assessed the acceptance of neonate *A. argillacea* larvae to Bt and non-Bt cotton plants at different temperatures during the first 24 h after hatching. Two cotton cultivars were used in the study, one a Bt DP 404 BG (Bollgard) cultivar, and the other, an untransformed isoline, DP 4049 cultivar. There was a greater acceptance by live neonate *A. argillacea* larvae for the non-Bt cotton plants compared with the Bt cotton plants, especially in the time interval between 18 and 24 h. The percentages of neonate *A. argillacea* larvae found on Bt or non-Bt plants were lower when exposed to temperatures of 31 and 34°C. The low acceptance of *A. argillacea* larvae for Bt cotton plants at high temperatures stimulated the dispersion of *A. argillacea* larvae. Our results support the hypothesis that the dispersion and/or feeding behavior of neonate *A. argillacea* larvae is different between Bt and non-Bt cotton. The presence of the Cry1Ac toxin in Bt cotton plants, and its probable detection by the *A. argillacea* larvae tasting or eating it, increases the probability of dispersion from the plant where the larvae began. These findings may help to understand how the *A. argillacea* larvae detect the Cry1Ac toxin in Bt cotton and how the toxin affects the dispersion behavior of the larvae over time. Therefore, our results are extremely important for the management of resistance in populations of *A. argillacea* on Bt cotton.

## Introduction

Cotton leafworm *Alabama argillacea* (Hübner) (Lepidoptera: Noctuidae) is a species native to Southern and Central America, found in almost all cotton-growing regions, extending from southern Canada to northern Argentina [1].

In Brazilian cotton-growing regions, this pest can infest the crop at any stage in its phonological development [2]. In Southern–Central Brazil, it is considered a late pest [3], but in the Northeast, with the exception of Bahia State, it attacks in the initial stages and can occur sporadically when the crop has matured [2]. The cotton leafworm, is highly destructive as one of the main defoliating pests of cotton (*Gossypium hirsutum Linnaeus*) in Brazil [4]. The most severe attacks occur after the cotton flowering period and are characterized by the destruction of the leaves on the plant's main stem, which reduces plant growth at any further stage of its development by affecting the height and the stem diameter, and production is damaged [5]. A high-density infestation can adversely affect the cotton yield. In Brazil, losses caused by *A. argillacea* vary from 21 to 35% of the cotton lint yield [1]. Chemicals are the main method for control; however, insect resistance to these molecules has been detected [6].

As an alternative to the currently used chemical control methods, cotton cultivars resistant to the cotton leafworm can be used to minimize the damage caused by this pest in cotton growing areas. The bacteria *Bacillus thuringiensis* subsp. *kurstaki* expresses protein crystals (Cry) during sporulation, and genetically modified plants with genes from the bacteria (Bt) express these protein crystals (Cry) that are deadly when ingested by lepidopteran larvae [7], [8].

The high efficiency of Bt cotton against lepidopterans has contributed to this technology being adopted quickly by cotton farmers in Brazil's Cerrado region, making it a specific tactic to protect cotton from *A. argillacea* damage [9]. However, the monophagous diet of *A. argillacea* on cotton and its high degree of susceptibility to Cry toxins may place selection pressure on populations of this insect. Consequently, the insect could alter its host selection behavior [10], [11] and accelerate the development of resistance, effectively jeopardizing the control of the pest and the income of the farm [12].

Insect resistance management (IRM) refers to a set of practices applied to prevent or delay the development of pest resistance to the methods used for its control [13] in agricultural areas. In view of IRM, the best way to preserve the benefits of transgenic plants is to establish refuge areas, and configure planting so blocks of non-Bt cotton are in areas adjacent to Bt cotton planting areas [9]. However, the use of this tactic can be inconvenient for the producer because it involves an additional cost in time, labor and seeds, which may contribute to its failure being accepted by cotton growers [14].

In an attempt to slow down the development of insect resistance to Bt toxins [13], [15], the main multinational seed-producing companies have considered the possibility of mixing a percentage of nontransgenic seeds directly into bags with transgenic seeds [13], to facilitate compliance with the technical standards to implement refuges. However, a concern about the use of this tactic is that the larvae, with some level of tolerance to Bt cotton, begins to feed on non-Bt cotton plants, but then disperses and feeds on Bt cotton plants, and survives, reaches adulthood and produces offspring with partial resistance to Bt toxins [16]. Another concern is that some newly hatched larvae are able to feed on Bt cotton plants, but then migrate to a nearby non-Bt cotton plant, thereby surviving to adulthood to produce offspring with partial resistance to Bt toxins [17]. Both scenarios increase the likelihood that heterozygous individuals will survive and potentially accelerate the development of resistance [18].

High relative humidity and temperatures decreased the effects of the Bt toxin, which does not occur at low temperatures, where there is a significant increase in the production of this toxin [19]. Therefore, in areas where the relative humidity and temperature are high, the efficiency of the Cry protein is possibly reduced, affecting the feeding behavior of the target pest, and consequently, resistance development. Thus, information on the feeding behavior and on the host acceptance of newly hatched cotton leafworm larvae for Bt cotton plants is essential. Information on *A. argillacea* populations and their potential dispersal and development of resistance to Bt cotton is necessary to determine whether a mixture of seeds is a viable management option compared with other tactical refuges to delay the evolution of resistance in *A. argillacea* populations. Thus, the aim of this study was to characterize and compare the degree of host acceptance of newly hatched *A. argillacea* larvae on Bt and non-Bt cotton plants at different temperatures during the first 24 h after hatching. Neonate larvae were used in these bioassays because they are very mobile and accept the host plant better than the later stages [20]. We proposed two hypotheses: a_1_) a higher percentage of neonate *A. argillacea* larvae would feed on non-Bt cotton plants than on Bt cotton plants and a_2_) after feeding started on the cotton plants, a higher percentage of neonate *A. argillacea* larvae would disperse from Bt cotton plants than from non-Bt cotton plants. The knowledge generated in this study will be important to develop a more effective management tactic to prevent *A. argillacea* population resistance to Bt cotton.

## Materials and Methods

### Insects and cotton cultivars

*Alabama argillacea* was grown at the Embrapa Cotton Entomology Laboratory, Biological Control Unit (UCB), Campina Grande, PB, Brazil. Larvae rearing stock were kept in a climate-controlled chamber at 25°C, with a relative humidity of 70±10% and 12-h photoperiod.

Two cotton cultivars were used in the study, one a Bt DP 404 BG (Bollgard) cultivar, and the other, an untransformed isoline, DP 4049 cultivar. The Bt and non-Bt cotton cultivars were planted separately in plastic pots (20 cm in diameter and 30 cm in height) and kept in a greenhouse.

### Bioassays

#### Dispersion behavior of neonate larvae at different temperatures and over time

To assess the effect of Bt and non-Bt cotton cultivars on the dispersion behavior of *A. argillacea* larvae at different temperatures and over time, plants from each (Bt and non-Bt) cotton cultivar that reached the eight-leaf stage received a newly hatched larva (0–24 hours old) released on a leaf in the plant's apical region. Then, each plant was covered with an organza bag and placed randomly in climate-controlled chambers at 22, 25, 28, 31 and 34°C, with a relative humidity of 70±10% and a photoperiod of 12 h. Daily the plants were moved randomly to minimize position effects within the chamber.

The experiment used was a 2×5×4 factorial in randomized blocks, where each block was divided into five parts (temperatures) and subdivided into four time intervals for assessment at 6, 12, 18 and 24 hours after plant infestation. Each subpart consisted of five replications, each with eight plants (four Bt cotton and four non-Bt cotton). After each time interval, the cotton plants were inspected and the larvae were removed with a brush. The larvae were categorized according to their location: found on the plant or on the organza bag.

### Neonate larvae feeding behavior over time

This test was conducted to quantify the percentages of neonate *A. argillacea* larvae that fed on Bt and non-Bt cotton plants. The experiment used was a 2×4 factorial in randomized blocks, with two cotton cultivars (Bt and non-Bt cotton) and four periods of plant exposure to larvae, i.e., 6, 12, 18 and 24 h after plant infestation. The experimental unit consisted of a Bt or non-Bt cotton plant cultivar that reached the eight-leaf stage and received 30 neonate *A. argillacea* larvae (0-24-h-old) released onto a leaf at the apical region of the plant. Then, each plant was covered with an organza bag and placed randomly in climate-controlled chambers at 28°C, with a relative humidity of 70±10% and photoperiod of 12 h. Daily the plants were moved randomly to minimize position effects within the chamber. After each time interval, the cotton plants and the inside of the organza bags were inspected and the larvae were removed with a brush. Then, the larvae were grouped into two categories: found on the plants or on the inside of the organza bags. To check that the larvae had fed, they were mounted on microscope slides in a solution of Karo honey diluted in water [21] and examined under a stereomicroscope (10×) according to the method adopted by Razze et al. [22]. The amount of plant material found in the gut of each caterpillar was measured by using an ocular micrometer attached to the microscope's eyepiece with phase contrast.

### Data analyses

The data from the bioassays were subjected to an analysis of variance (PROC GLM) [23] to determine whether there were effects of the cultivar (C), temperature (T), or time of exposure to cotton plants (t) on neonate *A. argillacea* larvae and to determine if the relationships between cultivar versus temperature, cultivar versus time and cultivar versus temperature versus time affected the percentage of neonate *A. argillacea* larvae recovered from the cotton plants. Additionally, the analysis of variance examined whether there was an interaction effect of cultivar (C) versus time of exposure to the cotton plants (t) on the percentage of neonate *A. argillacea* larvae that fed on Bt and non-Bt cotton cultivars at 28°C. An analysis of variance analysis was also performed (PROC GLM) [23] on the data collected for the percentage of larvae recovered at each location (plant or organza bag), on the amount of plant tissue recorded in the larvae's gut and to determine if there was an interaction between the cultivar and exposure time of the cotton plants to the larvae. The comparison of treatment means was performed using the Student-Newman-Keuls test (P = 0.05). A linear model (PROC CATMOD) [23] was used to estimate the recovery of *A. argillacea* larvae in Bt and non-Bt cotton plants depending on the temperature.

## Results

### Dispersion behavior at different time intervals and temperatures

There was a significant interaction between cotton cultivar (C) and the length of time *A. argillacea* larvae were exposed to the cotton plants (t) (F_(C versus t)3, 196_ = 10.29, P<0.0001) for the percentage of *A. argillacea* larvae recovered from the cotton plants (Table 1). Since P<0.05, there was significant interaction among cultivar (C), the length of time *A. argillacea* larvae were exposed to the cotton plants (t) and temperature (T). However, there were no interaction effects between cultivar (C) and temperature (T) (F_(C *versus* T_)_4, 196_ = 1.53, P = 0.1967) or between the length of time *A. argillacea* larvae were exposed to the cotton plants (t) and temperature (T) (F_(t *versus* T)12, 196_ = 0.99, P = 0.4595) in the percentage of recovered *A. argillacea* larvae from the cotton plants (Table 1). Therefore, the effects of cotton cultivar (Bt or non-Bt) on the percentage of recovered *A. argillacea* larvae from cotton plants depended on the length of time *A. argillacea* larvae were exposed to the cotton plants and the temperature.

**Table 1:** Summarized model of the three-way analysis of variance (ANOVA) for the effects of cultivar^1^, exposure time interval of neonate larvae to Bt cotton or non-Bt cotton^2^, and temperature^3^ on the percentage of neonate larvae of *A. argillacea* recovered from Bt cotton and non-Bt near isoline cotton plants.

Source	Models	DF	F ratio	Prob> F
Percentage of cotton leafworm	Model	43	6.32	0.0001
larvae recovered from cotton plant				
	Cultivar (C)	1	151.75	0.0001
	Time (t)	3	2.12	0.0997
	Temperature (T)	4	8.53	0.0001
	C *x* t	3	10.29	0.0001
	C *x* T	4	1.53	0.1967
	t *x* T	12	0.99	0.4595
	C *x* t *x* T	12	2.00	0.0274

1 Cultivars: Bt cotton and non-Bt near isoline cotton.

2 Time intervals: 0–6 h, 6–12 h, 12–18 h, and 18–24 h.

3 Temperatures (°C): 22, 25, 28, 31, and 34. Analysis was performed with the data transformed with arcsine square root percentage.

According to the analysis of variance, significant differences were found between cultivars Bt and non-Bt (F_(C)1, 196_ = 151.75, P<0.0001) (Table 1) and among temperatures tested (T) (F_(T)4, 196_ = 8.53, *P*<0.0001) (Table 1), but not for the exposure times of *A. argillacea* larvae to the cotton plants (t) (F_(t)3, 196_ = 2.12, *P* = 0.0997) (Table 1) in the percentage of recovered *A. argillacea* larvae from the cotton plants. However, there was no significant difference between the cotton cultivars (Bt or non-Bt) for the percentage of *A. argillacea* larvae recovered from the cotton plants in the first 6 h after release (Table 2). In the other time intervals, however, the Bt cotton cultivar had a significant reduction in the percentage of *A. argillacea* larvae recovered from the cotton plants when compared with the non-Bt plants, which was most evident in the time interval between 18 and 24 h (Table 2).

**Table 2:** Mean (± SE) percentage of neonates recovered from the cotton plants in the test for abandonment of neonate larvae of *A. argillacea* from Bt and non-Bt cotton plants during four exposure time intervals (F_(C x t) 3, 196_ = 10.29, P<0.00001).

Exposure time interval of neonate larvae to cotton plants (h)	Cultivar^1^	
	Bt cotton	Non-Bt cotton
0–6	83.08±12.96 Aa	89.57±7.07 Aa
6–12	75.49±16.47 Bb	93.60±8.48 Aa
12–18	71.36±17.69 Bb	93.05±8.46 Aa
18–24	62.26±23.40 Cb	94.07±8.09 Aa

1 Means within the same cultivar column with the same capital letters or means between cultivars within the same row with the same lower case letters are not significantly different (P = 0.05, Student-Newman-Keuls test). Original data.

### Dispersion behavior after 24 h

For the live and dead *A. argillacea* larvae recovered from the Bt and non-Bt cotton plants, the percentage of live *A. argillacea* larvae recovered from the cotton plants was significantly affected by the cotton cultivar (Bt and non-Bt) (F_(C)1, 36_ = 127.78, P<0.0001) (Table 3) and by the temperature (F_(T)4, 36_ = 7.81, P<0.0001) (Table 3), while the percentage of dead *A. argillacea* was only affected by the cotton cultivar (F_1, 36_ = 19.84, P<0.0001) (Table 3). The cultivar versus temperature interaction was not significant for either the percentage of live *A. argillacea* recovered from cotton plants (F_(C *versus* T)4, 36_ = 0.98, P = 0.4320) (Table 3) or the percentage of dead *A. argillacea* recovered (F_(C *versus* T)4, 36_ = 2.06, P = 0.0610) (Table 3). Therefore, the effect of the cotton cultivar on the percentage of dead or alive *A. argillacea* larvae recovered from the cotton plants did not depend on temperature.

**Table 3:** Summarized model of the two-way analysis of variance (ANOVA) for the effects of cultivar^1^ and temperature^2^ on the percentage of neonate larvae of *A. argillacea* recovered alive or dead from Bt cotton or non-Bt near isoline plants after 24 h.

Source	Models	DF	F ratio	Prob> F
Neonate larvae of *A. argillacea*	Model	13	12.74	0.0001
recovered alive from cotton plant				
(%)^3^ after 24 h				
	Cultivar (C)	1	127.78	0.0001
	Temperature (T)	4	7.81	0.0001
	C x T	4	0.98	0.4320
Neonate larvae of *A. argillacea*	Model	13	3.15	0.0032
recovered dead from cotton plant				
(%)^3^ after 24 h				
	Cultivar (C)	1	19.84	0.0001
	Temperature (T)	4	1.94	0.1250
	C x T	4	2.06	0.0610

1 Cultivars: Bt cotton and non-Bt near isoline cotton. ^2^Temperatures (°C): 22, 25, 28, 31, and 34. ^3^Data were arcsine-square root transformed prior to statistical analyses.

**Figure 1:**
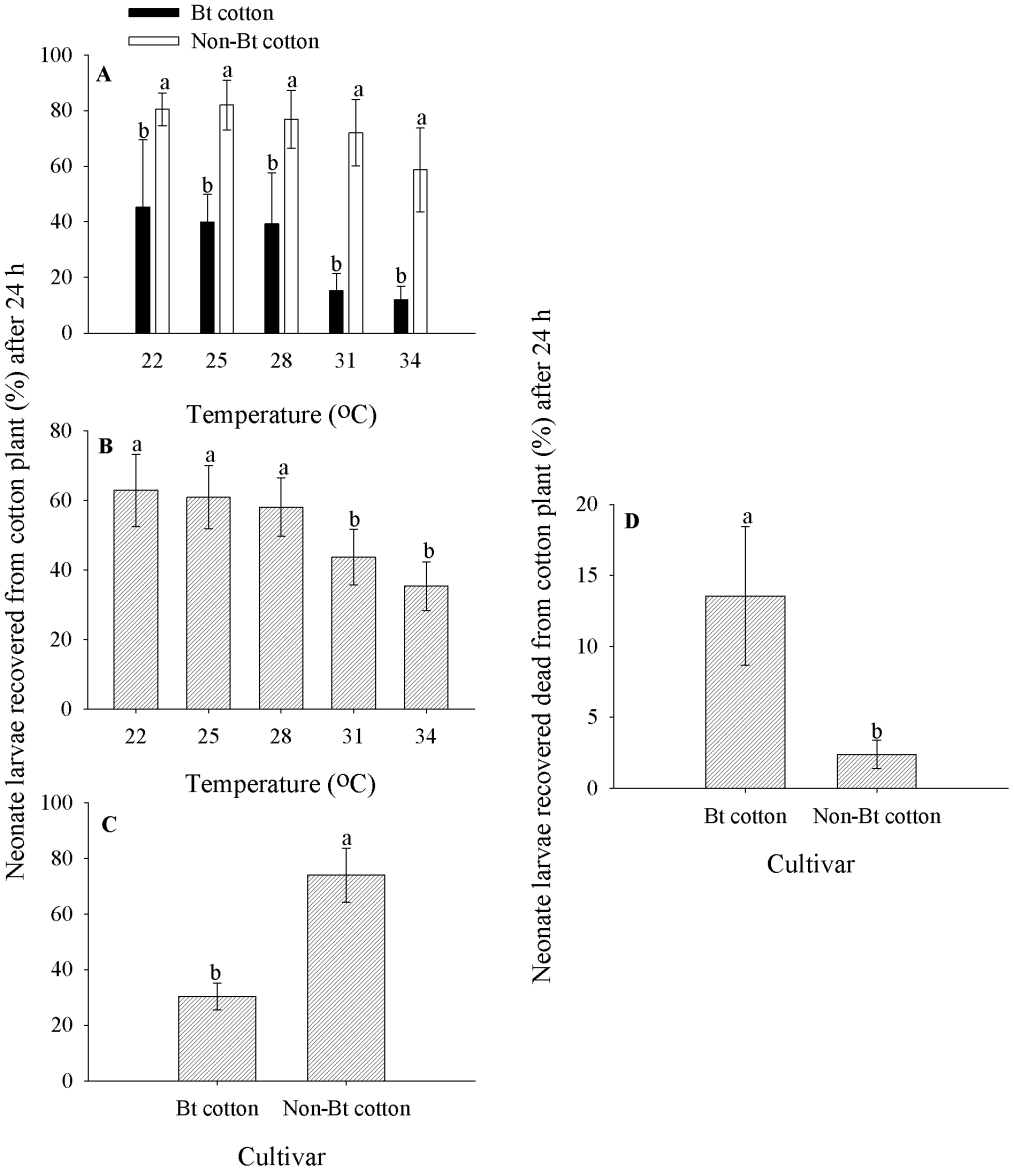
Mean percentage (± SE) of neonate larvae of *A. argillacea* recovered from cotton plants after 24 h. **A**. Bt and non-Bt cotton plants at each temperature (means followed by the same letter within each temperature are not significantly different by the Student-Newman-Keuls test, P = 0.05), **B**. Neonate larvae recovered alive from the cotton plants (means followed by the same letter are not significantly different by the Student-Newman-Keuls test, P = 0.05), **C**. Bt and non-Bt cotton plants (F_(C)1, 36_ = 127.78, P<0.0001), and **D**. Neonate larvae recovered dead from Bt and non-Bt cotton plants after 24 h (F_(C)1,36_ = 19.84, P<0.0001). Original data.

The percentage of live *A. argillacea* larvae recovered from the cotton plants after 24 h, at each of the temperatures, was significantly higher in the non-Bt cotton cultivar than in the Bt cotton cultivar (Fig. 1A). Regarding the influence of temperature, the percentage of live *A. argillacea* larvae recovered from the cotton plants was lower at 31 and 34°C than at the other temperatures, and there were no significant differences among the other temperatures (Fig. 1B). When summed across all temperatures, the percentage of live *A. argillacea* larvae recovered from the Bt cotton plants after 24 h was significantly lower than those recovered from the non-Bt cotton plants (Fig. 1C). The percentage of dead *A. argillacea* larvae recovered from the cotton plants after 24 h, summed across all temperatures, was significantly higher in the Bt cotton cultivar than in the non-Bt cotton cultivar (Fig. 1D).

**Figure 2:**
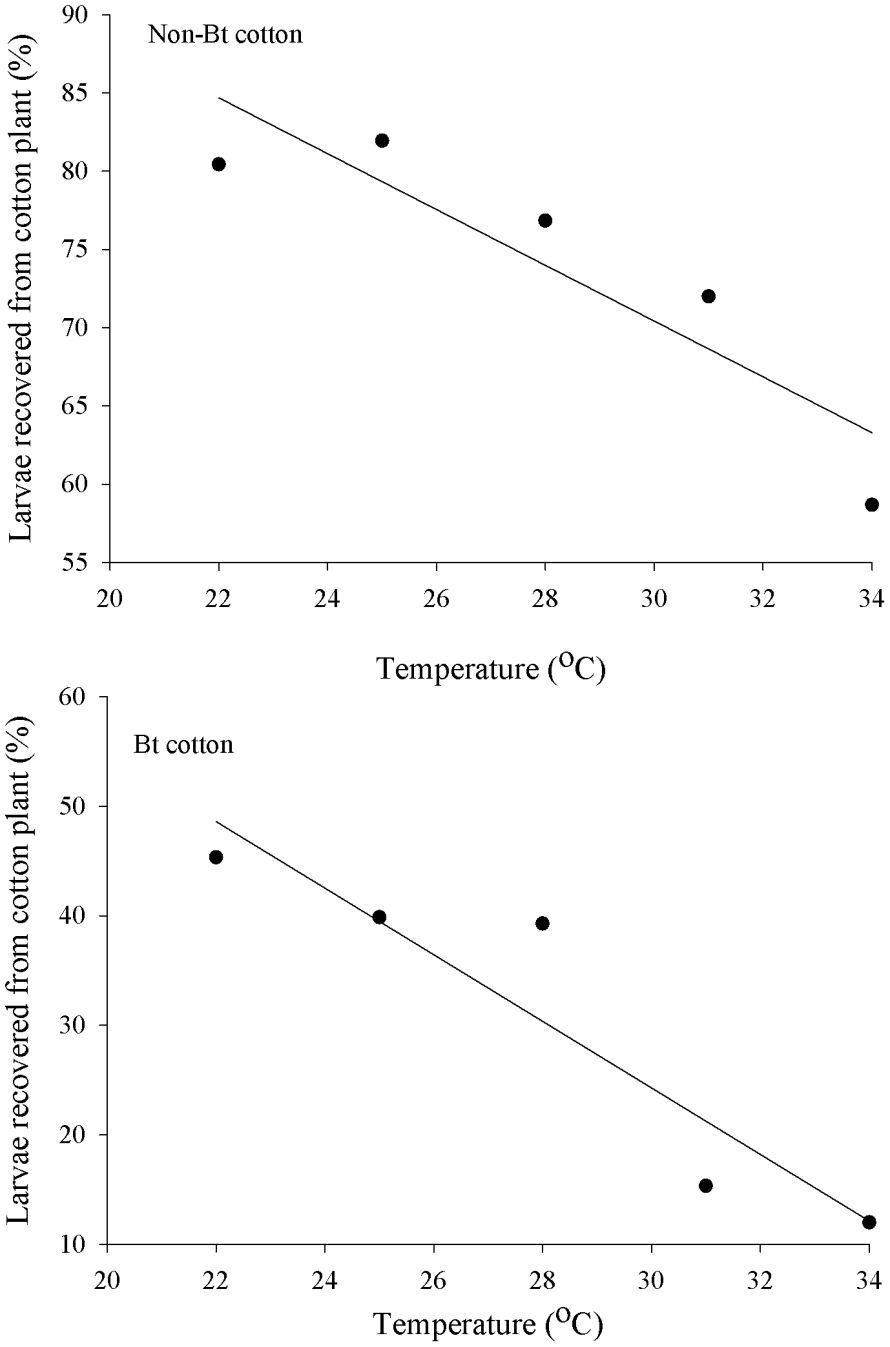
Relationship between the mean percent of neonate larvae of *A. argillacea* recovered from plants of Bt cotton (y = 115.47 – 3.04x, R^2^ = 0.87, F_1, 3_ = 19.90, P<0.0210) and non-Bt cotton (y = 123.90 – 1.78x, R^2^ = 0.81, F_1, 3_ = 13.09, P<0.0363) and temperature after 24 h. Original data.

A linear model best described the percentage of live *A. argillacea* larvae recovered from Bt or non-Bt cotton plants as a function of temperature (Fig. 2). The linear models showed that 81% and 87% of the variation for the average percentage of live *A. argillacea* larvae recovered from the non-Bt and Bt cotton plants, respectively, was explained by temperature (Fig. 2). However, the percentage of live *A. argillacea* larvae recovered from the cotton plants ranged from approximately 10.93% (34°C) to 48.60% (22°C) in Bt cotton plants, whereas it ranged from 63.80% (34°C) to 84.70% (22°C) in non-Bt cotton plants (Fig. 1A and Fig. 2).

### Feeding behavior in different time intervals

**Figure 3:**
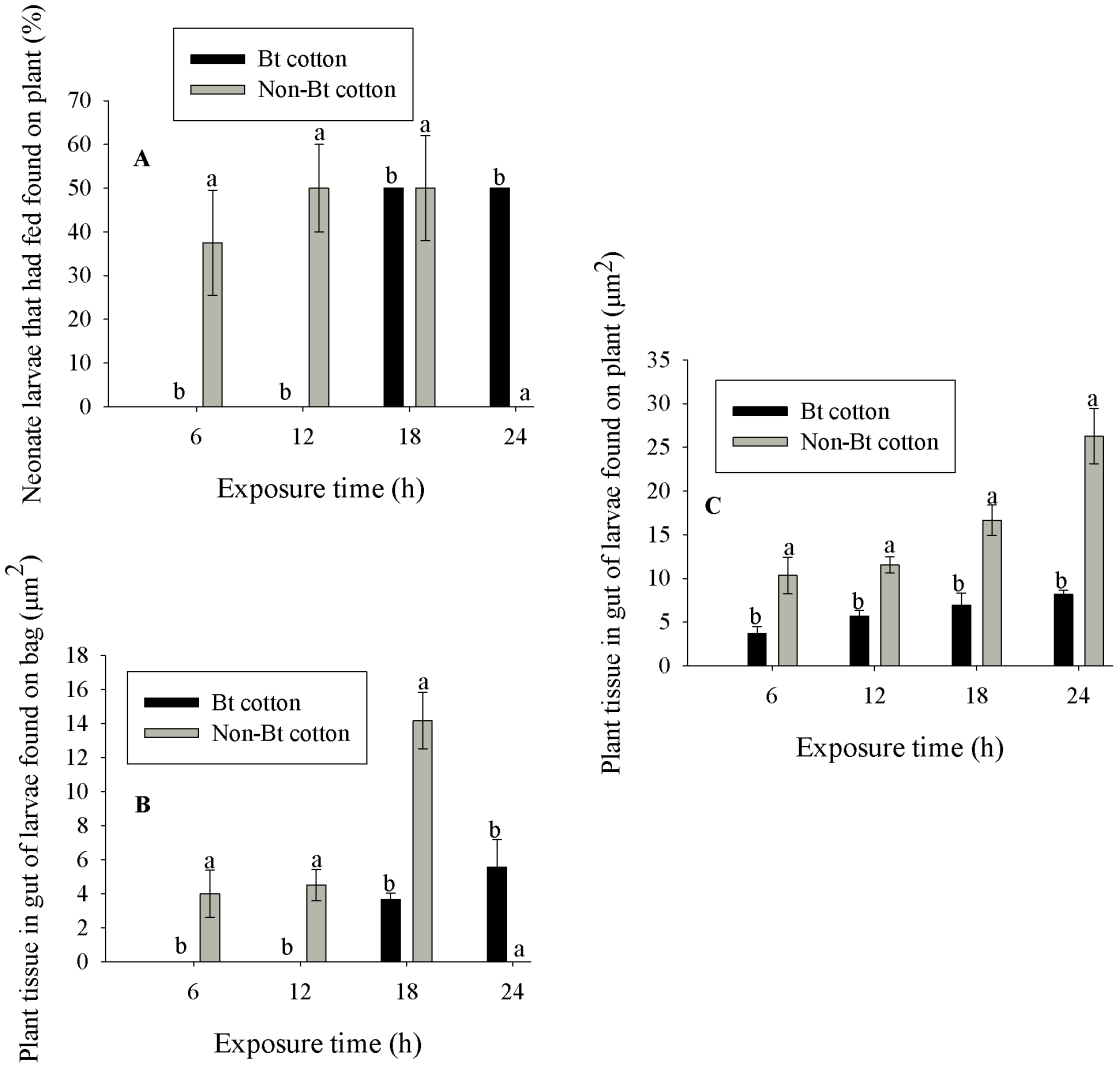
Mean percentage (± SE) of neonates of *A. argillacea* that had fed and were found on the plant on Bt and non-Bt cotton at 6, 12, 18 and 24 h (A), (B) mean gut values for plant tissue area (± SE) for larvae that fed and were found on the bag, or (C) on the Bt or non-Bt cotton plants at 28°C. Original data.

The percentages of neonate *A. argillacea* larvae that had fed and were found on the organza bag were affected significantly by the cotton cultivar (F_(C)1, 21_ = 5.70, P<0.0264), the exposure time (F_(Et)3, 21_ = 13.48, P<0.0001) and the cultivar versus exposure time interaction (F_(C *versus* Et)3, 21_ = 36.54, P<0.0001) (Table 4). However, the percentages of neonate *A. argillacea* larvae that had fed and were found on the plant were not affected by the cultivars (F_(C)1, 21_ = 0.01, P = 1.0000), the exposure time (F_(Et)3, 21_ = 0.64, P = 0.5999) or the cultivar versus exposure time interaction (F_(C *versus* Et)3, 21_ = 1.27, P = 0.3094) (Table 4). The average percentage of larvae that had fed and were on the organza bag was higher in the non-Bt cotton cultivar than in the Bt cotton cultivar (Fig. 3A). The percentages of larvae that had fed and that were found on the organza bag increased over exposure time for both cotton cultivars (Bt and non-Bt) (Fig. 3A).

**Table 4:** Summarized model of the two-way analyses of variance (ANOVA) for the effects of cultivar^1^ and exposure time^2^ on the percentage of neonate larvae of *A. argillacea* that had fed and were found on the plant or the bag (Bt or non-Bt near isoline)^3^ and on the amount of plant tissue in the gut of neonate larvae of *A. argillacea* that were found on the plant or the bag at 6, 12, 18, and 24 h.

Source	Models	DF	F ratio	Prob> F
Neonate larvae of *A. argillacea* that had	Model	10	0.76	0.6609
fed that were found on the plant (%)				
	Cultivar (C)	1	0.01	1.0000
	Exposure time (Et)	3	0.64	0.5999
	C x Et	3	1.27	0.3094
Neonate larvae of *A. argillacea* that had	Model	10	15.96	0.0001
fed that were found on the bag (%)				
	Cultivar (C)	1	5.70	0.0264
	Exposure time (Et)	3	13.48	0.0001
	C x Et	3	36.54	0.0001
Plant tissue amount in the gut of neonate	Model	10	15.79	0.0001
larvae of *A. argillacea* that were found				
on the plant (μm^2^)				
	Cultivar (C)	1	95.69	0.0001
	Exposure time (Et)	3	16.65	0.0001
	C x Et	3	2.97	0.0550
Plant tissue amount in the gut of neonate	Model	10	19.77	0.0001
larvae of *A. argillacea* that were found				
on the bag (μm^2^)				
	Cultivar (C)	1	25.25	0.0001
	Exposure time (Et)	3	25.51	0.0001
	C x Et	3	30.90	0.0001

1 Cultivars: Bt cotton and non-Bt near isoline cotton. ^2^Time: 6, 12, 18, and 24 h. ^3^Data were arcsine-square root transformed prior to statistical analyses.

The quantities of plant tissue measured in the intestines of the neonate *A. argillacea* larvae that fed and were found on the plant or in the organza bag were affected by the cotton cultivar (plant: F_(C)1, 21_ = 95.69, P<0.0001; organza bag: F_(C)1, 21_ = 25.25, P<0.0001), the exposure time (plant: F_(Et)1, 21_ = 16.65, P<0.0001; organza bag: F_(C)1, 21_ = 25.51, P<0.0001) and the cultivar versus exposure time interaction (plant: F_(C *versus* Et)3, 21_ = 2.97, P = 0.0550; organza bag: F_(C *versus* Et)3, 21_ = 30.90, P<0.0001). The average amount of plant tissue found in the gut of the fed *A. argillacea* larvae, found on the plant or in the organza bag, was higher for non-Bt cotton plants than Bt plants (Figs. 3B and 3C), except at 24 h after infestation by the larvae found in the organza bag (Fig. 3B). These values increased with exposure time for both Bt cotton plants and non-Bt plants (Figs. 3B and 3C).

## Discussion

The dispersion of neonate lepidopteran pest larvae can be the result of genetic programming to reduce competition for resources and ensure the survival of the larvae [17], [22]. The dispersal of *A. argillacea* larvae on Bt and non-Bt cotton plants could be related to host plant acceptance, suggesting that the *A. argillacea* larvae are more likely to abandon Bt plants than non-Bt plants, which would result in less feeding on Bt plants. The percentage of live neonate *A. argillacea* larvae recovered from the non-Bt cotton cultivars was significantly higher than the percentage of *A. argillacea* larvae recovered from the Bt cotton cultivars. The one exception was in the initial interval 6 h after the neonate *A. argillacea* larvae were released onto the cotton plants, because there was no significant difference between Bt and non-Bt cotton cultivars in larvae recovered (Table 2). Razze et al. [22] reported that over 95% of neonate *Ostrinia nubilalis* (Hübner) (Lepidoptera: Cambridae) larvae who left the maize plants (*Zea mays* Linnaeus) in the first 6 h after they were exposed to the maize plants (Bt and non-Bt corn) fed very little or did not feed, regardless of whether the plants were Bt or non-Bt corn cultivars. Goldstein et al. [17] found that significantly more neonate larvae of *O. nubilalis* present on non-Bt than on Bt corn 24 h after blackhead egg masses were placed on the plants.

We found a higher percentage of neonate *A. argillacea* larvae recovered from the organza bags with evidence that they fed on the Bt cotton cultivar compared with the non-Bt cultivar after 24 h of infestation (Fig. 3A). According to Razze et al. [22] after 48 h, there was a significantly higher percentage of *O. nubilalis* larvae that had evidence of feeding that were found on the bag on Bt corn compared with non-Bt near isoline.

This, combined with less plant material found in the intestines of larvae from the Bt plants compared with the non-Bt plants (Figs. 3B and 3C), may explain how *A. argillacea* responds to the Cry1Ac protein in cotton plants. Larvae at the start of the test fed on Bt cotton plants and realized that it was not palatable and likely sought to leave the Bt plant for a more suitable host plant. If the larvae had located another Bt plant, it would most likely try to feed and again find that the plant was not palatable and so attempt to leave the plant. This could be repeated several times. If the larva was constantly exposed to Bt cotton plants, the larva could try feeding several times; however, the larvae would not accumulate much plant tissue in its intestine. On the other hand, as the Cry1Ac protein is harmful to the insects, Bt-cotton might disturb the digestion of the plant material and contribute to the accumulation of the plant material in the gut. Our results demonstrated that after 18 h the amount of plant tissue found in the intestines of larvae exposed to Bt plants was less than that recorded in the intestines of larvae exposed to non-Bt plants, both for the larvae recovered on the plant (Fig. 3C) and for the larvae recovered on the organza bag (Fig. 3B). According to Davis and Coleman [24], *O. nubilalis* larvae will rarely stop feeding when continually exposed to a Bt plant.

It is likely that a percentage of individuals produced by *A. argillacea* moths are genetically programmed to disperse from the cotton plants without feeding on the host. One of the advantages of neonate *A. argillacea* larvae immediately migrating from the host plant is to escape interspecific competition to increase its survivability. According to Schultz and Baldwin (1982) [25], *Lymantria dispar dispar* larvae (Linnaeus) (Lepidoptera: Erebidae) can induce changes in *Quercus* spp. leaves, which may make them less palatable for subsequent herbivores. Additionally, the dispersion of some neonate *A. argillacea* larvae from cotton plants to other host plants can be a selective advantage for some larvae to avoid predators and parasitoids that are attracted to the aggregation of the neonate *A. argillacea* larvae. Goldstein et al. [17] and Razze et al. [22] suggested that various behaviors of neonate *O. nubilalis* larvae hatched in a mass of eggs were genetically controlled.

This dispersion behavior displayed by the *A. argillacea* larvae on cotton plants can have various consequences, such as contributing to the survival of the target pest (*A. argillacea*). Hence, dispersion behavior may reduce the effectiveness of strategies, such as mixing transgenic and nontransgenic seeds, to manage and delay lepidopteran pests from developing resistance to the Bt cotton cultivars. Moreover, the early migration of neonate *A. argillacea* larvae stimulated by the Bt cotton cultivars compared with non-Bt plants does not prevent larvae in more developed instars, which are more tolerant to Bt proteins, from migrating later from non-Bt cotton plants to Bt cotton plants; thus, favoring the development of resistance in lepidopteran pests to Bt cotton cultivars [26].

One of the main environmental risks associated with Bt crops is the potential for populations of target pests to develop resistance to Bt proteins, where the pests are controlled using this technology [12]. Cases of lepidopteran pests becoming resistant to Bt cotton cultivars were detected for *Pectinophora gossypiella* (Saunders) (Lepidoptera: Gelechiidae) [27] and *Helicoverpa armigera* (Hübner) (Lepidoptera: Noctuidae) [28]. Larvae that have emerged on a Bt plant can test, stop eating, and disperse to a more acceptable host [22].

The results of our study show that 6 h after the neonate *A. argillacea* larvae were released on Bt and non-Bt cotton cultivar plants, especially between 18 and 24 h (Table 2), the *A. argillacea* larvae acceptance rate of Bt cotton cultivar plants was significantly lower than for non-Bt cotton cultivar plants. This behavior shown by the neonate *A. argillacea* larvae suggests that in the first 6 h they are exposed to the cotton plants, they are not able to identify if the cotton cultivar is Bt or non-Bt; however, identification occurs between 6-12 h after it comes in contact with the plants.

The high susceptibility of neonate *A. argillacea* larvae to the Bt toxin may have induced the dispersal of larvae in a significantly higher percentage in the Bt cotton cultivar compared with non-Bt cotton cultivar. According to Razze et al. [22] and Davis and Onstad [29], newly hatched *O. nubilalis* larvae dispersed more quickly on Bt corn plants than on non-Bt corn plants. Similar behavior was reported by López et al. [26] for *Sesamia nonagrioides* Lefebvre (Lepidoptera: Noctuidae) larvae on Bt corn. According to Goldstein et al. [17], the neonate *O. nubilalis* larvae were able to quickly detect Bt toxins in the leaves of Bt corn plants. Thus, the behavior exhibited by the neonate *A. argillacea* larvae to quickly disperse from Bt cotton cultivar plants is most likely because they are able to quickly detect the presence of the Bt toxin in the Bt cotton cultivar plants. However, an increase in dispersal of larvae caused by Bt plants may result in a lower efficiency for the refuge strategy [26]. Although *A. argillacea* is susceptible to the Cry1Ac toxin [30], [31], the risk of developing resistance to this toxin is high because populations of this Noctuidae are exposed to high selection pressure in Bt cotton.

The *A. argillacea* larvae remained on the Bt cotton generally survived because the mean percentage of neonate larvae recovered dead from the plant was <15% (Fig. 1D), but the survival rate was about 30% (Fig. 1C); *A. argillacea* larvae mortality was significantly higher on Bt cotton cultivar plants than on non-Bt cotton cultivar plants (Fig. 1D). Similar results were found by López et al. [26] for *S. nonagrioides* larvae on Bt corn plants. Sousa et al. [30] reported a mortality of 90% of *A. argillacea* larvae that fed on Bt cotton leaves, 1 h after ingestion.

A differentiation in an *A. argillacea* larvae's acceptance of Bt cotton cultivar plants compared with non-Bt cotton cultivar plants may result in greater selection pressure on *A. argillacea* populations for resistance to Bt toxins. For example, if the mixed seed strategy was used, *A. argillacea* larvae could disperse to the nearest non-Bt cotton plant or migrate to structures with lower toxin concentrations, such as the bud bracts [32]. Therefore, the behavior of *A. argillacea* larvae not accepting the Bt cotton plants as food is important, and it should be considered in decisions on managing the resistance of *A. argillacea* to the Cry1Ac toxin.

For Brazilian cotton farmers, refuge-in-a-bag tactics can provide some advantages because there is no need to plan structured refuge areas (block or strip plantings), and the lack of adoption of standard measures in refuge areas, such as intensive use of chemicals to control target and non-target arthropods on genetically modified plants, can be avoided. Logistically, the refuge-in-a-bag tactic could be of paramount importance for Brazilian producers. This approach could make at least one resistance management tactic be adopted, the use of structured refuges, which in Brazil has received little attention for various reasons. However, the refuge-in-a-bag tactic might accelerate the development of resistance to Bt toxins in *A. argillacea*. Moreover, according to population simulations by Mallet and Porter [18], the refuge-in-a-bag tactic is not an effective measure to slow the development of resistance; paradoxically, this refuge tactic may even contribute to resistance developing, especially in situations where the level of dominance is approximately 0.01 and under conditions where the probability to select resistant individuals is high. Considering that the high dispersal of *A. argillacea* larvae within the “patch” also tends to cause an effective dominance, it is likely that an *A. argillacea* larva that feeds on a non-Bt cotton cultivar plant can grow and then migrate to and damage a Bt cotton cultivar plant and, thus, heterozygous individuals can still survive. To implement this measure, individuals must be crossed from both situations, i.e., a certain synchronicity of insect emergences from Bt and non-Bt cotton plants.

Another disadvantage of adopting the refuge-in-a-bag tactic, aimed at managing pest resistance in Brazil, is the scarcity of information on the mobility of target pests on transgenic plants, on the initial frequency of alleles that provide resistance, and on studies of the genetic structure of these populations [33]–[35].

High temperatures can influence the expression of the toxin in Bt crops. This can have several effects on the *A. argillacea* larvae's host acceptance behavior and the consequent dispersal and survival of *A. argillacea*. Studies have reported that high temperatures (36 to 40°C) can reduce Bt protein production in the Bt cotton plants, possibly inactivating the genes that express them, resulting in a lower efficiency of Bt plants against larvae during the open boll period [36]–[38]. However, this was not observed during vegetative growth or during flowering of the Bt cotton plants [36], [38]. Our study showed that the percentage of *A. argillacea* larvae recovered from the cotton plants after 24 h, regardless of the temperature, was significantly higher for the non-Bt cotton cultivar than for the Bt cotton cultivar. However, the percentage of neonate *A. argillacea* larvae recovered on cotton plants was lower at 31 and 34°C than at 22, 25 and 28°C, with no differences among the other temperatures. According to Medeiros et al. [39], *A. argillacea* larvae reached thermal stress at 33°C; it was assumed that at 35°C and above, the production of enzymes in *A. argillacea* larvae was partially inhibited [39]. In addition to the *A. argillacea* larvae's low acceptance of the host plant, the heat stress, regardless of the cultivars, may have stimulated the dispersion behavior of the neonate *A. argillacea* larvae. In general, the percentage of *A. argillacea* larvae found on Bt cotton plants was less than on non-Bt cotton plants.

The eggs, larvae and pupae of *A. argillacea* are attacked by natural enemies (entomopathogens, predators and parasitoids) [39]–[43], which together with abiotic factors contribute to a relatively high natural mortality [1]. Therefore, the probability of an *A. argillacea* larva dispersing from a Bt cotton plant to a non-Bt cotton plant and surviving is very low with the refuge-in-a-bag strategy. The efficiency of natural enemies in reducing populations of this pest in a structured refuge may be different from the refuge-in-a-bag tactic. The structured refuge may offer a more favorable environment for the development of natural enemies than the refuge-in-a-bag, especially for a structured refuge with a high concentration of *A. argillacea* on non-Bt cotton plants. However, the effect of the refuge configuration on the abundances of the populations of the natural enemies of *A. argillacea* (entomopathogens, predators and parasitoids) needs to be explored further, and cotton growers should consider establishing a mixed-seed-refuge.

Based on the percentage of neonate *A. argillacea* larvae recovered from the organza bags for Bt and non-Bt cotton cultivars, in the first 24 h that they were exposed to the plants, the dispersal of neonate *A. argillacea* larvae was quite high. Although the cotton plants had been protected by the organza bag and were under laboratory conditions, our results indicated that there were differences in the behavior of neonate *A. argillacea* larvae on Bt cotton plants compared with non-Bt cotton plants. After 24 h, a high percentage of *A. argillacea* larvae remained on the non-Bt cotton plants (Fig. 1A), while the dispersion of larvae from Bt cotton plants remained high (Fig. 1A). Similar results were found by Tang et al. [44] who studied the dispersion behavior of the third-instar larvae of *Plutella xylostella* (Linnaeus) (Lepidoptera: Plutellidae) on Bt and non-Bt broccoli (*Brassica oleracea* Linné) plants for a 72-h period. They found that when the release host was a Bt plant, most larval movement off the Bt plant occurred during the first 48 h with little change of movement between 48 and 72 h. When the release host was a non-Bt plant, all larval movement occurred during the first 24 h with little change of movement between 24 and 72 h. Most movement onto the second plant occurred within the first 24 h of the release with little movement occurring afterward [44]. These findings indicate that most of the cotton leafworm neonates are to detect the Bt endotoxins when exposed to the plant for 24 h and elicit behaviors leading to plant abandonment in response. If neonates abandoning Bt cotton are able to survive and find a more suitable host plant, there could be selection for behavioral resistance.

The results of our bioassays support the hypothesis that the dispersion behavior of neonate *A. argillacea* larvae is significantly different for Bt plants compared with non-Bt cotton plants. The presence of the Cry1Ac toxin in Bt cotton plants and its probable detection by the *A. argillacea* larvae tasting or feeding increases the probability of dispersion from the plant where they hatched. To understand the movement of *A. argillacea* larvae between Bt and non-Bt cotton plants and the likelihood of their survival after ingesting the Cry1Ac toxins, the relationships between their feeding behavior and their dispersion behavior need to be explored. Results obtained from the laboratory suggest that the last instar larvae of *O. nubilalis* can move from non-Bt corn plants to Bt corn plants and then survive until they reach adulthood [45], [46]; however, there was little evidence that this happened in the field. Therefore, in the case of cotton, more research is needed to determine the differences in feeding behavior and dispersal of *A. argillacea* larvae on Bt cotton plants compared to non-Bt plants in the field. Once this knowledge has been obtained, the effectiveness of the refuge tactic known as a refuge-in-a-bag can be determined as well as the risk of developing resistance in *A. argillacea* populations to Bt toxins. These findings may help to understand how the *A. argillacea* larvae detect the Cry1Ab toxin in Bt cotton and how it affects the dispersion behavior of the larvae over time. Therefore, our results are extremely important for the resistance management of *A. argillacea* populations on Bt cotton cultivars.

## Supporting Information

Data Set S1 **Data set for percentage of neonate larvare found on cotton plant.**
(DOCX)

Data Set S2 **Data set for neonate larvae recovered alive from cotton plant after 24 h.**
(DOCX)

Data Set S3 **Data set for neonate larvae recovered dead after 24 h.**
(DOCX)

Data Set S4 **Data set for neonate larvae that had fed found on plant and on bag (%).**
(DOCX)

Data Set S5 **Data set for plant tissue in gut of neonate larvae recovered from cotton plant.**
(DOCX)

Data Set S6 **Data set for plant tissue in gut of neonate larvae found on bag (μm2).**
(DOCX)
