# Supplementary material for: Feeding and Dispersal Behavior of the Cotton Leafworm, Alabama argillacea (Hübner) (Lepidoptera: Noctuidae), on Bt and Non-Bt Cotton: Implications for Evolution and Resistance Management
Source: PLoS One. 2014 Nov 4;9(11):e111588. doi: 10.1371/journal.pone.0111588 (PMC4219722; doi:10.1371/journal.pone.0111588)
Supplement: Data Set S1 — Data set for percentage of neonate larvare found on cotton plant. (DOCX) [file pone.0111588.s001.docx]

**Data Set S1.** Data set for percentage of neonate larvare found on cotton plant.

Temp = Temperature

Cult = Cultivar

Bloc = Block

Perc = Percentage

Bt = Bt cotton plant

Is = non-Bt cotton plant

Temp Cult Bloc Time Perc

22 Bt 1 6 100.00

22 Bt 1 12 100.00

22 Bt 1 18 91.67

22 Bt 1 24 100.00

22 Bt 2 6 86.21

22 Bt 2 12 65.52

22 Bt 2 18 64.29

22 Bt 2 24 78.57

22 Bt 3 6 100.00

22 Bt 3 12 100.00

22 Bt 3 18 96.43

22 Bt 3 24 95.65

22 Bt 4 6 96.43

22 Bt 4 12 96.15

22 Bt 4 18 100.00

22 Bt 4 24 95.83

22 Bt 5 6 90.00

22 Bt 5 12 80.00

22 Bt 5 18 80.00

22 Bt 5 24 66.67

25 Bt 1 6 85.71

25 Bt 1 12 91.30

25 Bt 1 18 90.48

25 Bt 1 24 85.71

25 Bt 2 6 88.89

25 Bt 2 12 91.67

25 Bt 2 18 100.00

25 Bt 2 24 100.00

25 Bt 3 6 92.86

25 Bt 3 12 100.00

25 Bt 3 18 100.00

25 Bt 3 24 100.00

25 Bt 4 6 100.00

25 Bt 4 12 95.65

25 Bt 4 18 95.24

25 Bt 4 24 94.12

25 Bt 5 6 93.33

25 Bt 5 12 100.00

25 Bt 5 18 100.00

25 Bt 5 24 50.00

28 Bt 1 6 92.31

28 Bt 1 12 100.00

28 Bt 1 18 90.91

28 Bt 1 24 93.33

28 Bt 2 6 93.33

28 Bt 2 12 84.21

28 Bt 2 18 100.00

28 Bt 2 24 91.67

28 Bt 3 6 82.76

28 Bt 3 12 100.00

28 Bt 3 18 100.00

28 Bt 3 24 100.00

28 Bt 4 6 89.29

28 Bt 4 12 83.33

28 Bt 4 18 86.96

28 Bt 4 24 65.22

28 Bt 5 6 100.00

28 Bt 5 12 100.00

28 Bt 5 18 91.67

28 Bt 5 24 95.65

28 Bt 6 6 92.59

28 Bt 6 12 100.00

28 Bt 6 18 94.74

28 Bt 6 24 100.00

28 Bt 7 6 96.30

28 Bt 7 12 94.12

28 Bt 7 18 94.12

28 Bt 7 24 100.00

31 Bt 1 6 100.00

31 Bt 1 12 100.00

31 Bt 1 18 100.00

31 Bt 1 24 100.00

31 Bt 2 6 100.00

31 Bt 2 12 100.00

31 Bt 2 18 100.00

31 Bt 2 24 100.00

31 Bt 3 6 100.00

31 Bt 3 12 100.00

31 Bt 3 18 100.00

31 Bt 3 24 0.00

31 Bt 4 6 88.24

31 Bt 4 12 85.71

31 Bt 4 18 100.00

31 Bt 4 24 85.71

31 Bt 5 6 95.24

31 Bt 5 12 95.00

31 Bt 5 18 90.00

31 Bt 5 24 60.00

34 Bt 1 6 100.00

34 Bt 1 12 100.00

34 Bt 1 18 80.00

34 Bt 1 24 50.00

34 Bt 2 6 90.00

34 Bt 2 12 100.00

34 Bt 2 18 87.50

34 Bt 2 24 100.00

34 Bt 3 6 100.00

34 Bt 3 12 100.00

34 Bt 3 18 85.71

34 Bt 3 24 71.43

34 Bt 4 6 100.00

34 Bt 4 12 82.61

34 Bt 4 18 100.00

34 Bt 4 24 100.00

34 Bt 5 6 86.67

34 Bt 5 12 95.24

34 Bt 5 18 91.67

34 Bt 5 24 100.00

22 IS 1 6 93.33

22 IS 1 12 96.67

22 IS 1 18 93.33

22 IS 1 24 82.14

22 IS 2 6 81.25

22 IS 2 12 92.59

22 IS 2 18 88.89

22 IS 2 24 62.96

22 IS 3 6 93.33

22 IS 3 12 86.67

22 IS 3 18 93.33

22 IS 3 24 83.33

22 IS 4 6 96.55

22 IS 4 12 96.55

22 IS 4 18 96.55

22 IS 4 24 89.66

22 IS 5 6 93.33

22 IS 5 12 73.33

22 IS 5 18 76.67

22 IS 5 24 73.33

25 IS 1 6 80.00

25 IS 1 12 96.67

25 IS 1 18 96.67

25 IS 1 24 96.67

25 IS 2 6 89.66

25 IS 2 12 93.10

25 IS 2 18 96.55

25 IS 2 24 100.00

25 IS 3 6 86.67

25 IS 3 12 100.00

25 IS 3 18 100.00

25 IS 3 24 100.00

25 IS 4 6 73.33

25 IS 4 12 100.00

25 IS 4 18 96.55

25 IS 4 24 100.00

25 IS 5 6 89.29

25 IS 5 12 100.00

25 IS 5 18 100.00

25 IS 5 24 100.00

28 IS 1 6 88.89

28 IS 1 12 100.00

28 IS 1 18 96.30

28 IS 1 24 100.00

28 IS 2 6 93.55

28 IS 2 12 100.00

28 IS 2 18 90.32

28 IS 2 24 96.77

28 IS 3 6 93.33

28 IS 3 12 100.00

28 IS 3 18 100.00

28 IS 3 24 92.00

28 IS 4 6 93.33

28 IS 4 12 93.33

28 IS 4 18 86.67

28 IS 4 24 96.67

28 IS 5 6 96.67

28 IS 5 12 93.10

28 IS 5 18 89.66

28 IS 5 24 86.21

28 IS 6 6 93.33

28 IS 6 12 93.33

28 IS 6 18 96.67

28 IS 6 24 100.00

28 IS 7 6 100.00

28 IS 7 12 100.00

28 IS 7 18 100.00

28 IS 7 24 100.00

31 IS 1 6 100.00

31 IS 1 12 93.33

31 IS 1 18 100.00

31 IS 1 24 95.45

31 IS 2 6 100.00

31 IS 2 12 100.00

31 IS 2 18 100.00

31 IS 2 24 100.00

31 IS 3 6 100.00

31 IS 3 12 96.43

31 IS 3 18 100.00

31 IS 3 24 100.00

31 IS 4 6 100.00

31 IS 4 12 100.00

31 IS 4 18 90.91

31 IS 4 24 95.45

31 IS 5 6 88.89

31 IS 5 12 95.00

31 IS 5 18 90.00

31 IS 5 24 95.00

34 IS 1 6 100.00

34 IS 1 12 92.59

34 IS 1 18 100.00

34 IS 1 24 100.00

34 IS 2 6 100.00

34 IS 2 12 89.47

34 IS 2 18 84.21

34 IS 2 24 84.21

34 IS 3 6 96.43

34 IS 3 12 100.00

34 IS 3 18 100.00

34 IS 3 24 92.31

34 IS 4 6 100.00

34 IS 4 12 90.00

34 IS 4 18 100.00

34 IS 4 24 100.00

34 IS 5 6 83.33

34 IS 5 12 100.00

34 IS 5 18 100.00

34 IS 5 24 100.00
