# Supplementary material for: Feeding and Dispersal Behavior of the Cotton Leafworm, Alabama argillacea (Hübner) (Lepidoptera: Noctuidae), on Bt and Non-Bt Cotton: Implications for Evolution and Resistance Management
Source: PLoS One. 2014 Nov 4;9(11):e111588. doi: 10.1371/journal.pone.0111588 (PMC4219722; doi:10.1371/journal.pone.0111588)
Supplement: Data Set S2 — Data set for neonate larvae recovered alive from cotton plant after 24 h. (DOCX) [file pone.0111588.s002.docx]

**Data Set S2.** Data set for neonate larvae recovered alive from cotton plant after 24 h.

Cult = Cultivar

Temperat = Temperature

Block = Block

Perc24h = Percentage – 24 h

Bt = Bt cotton plant

Is = non-Bt cotton plant

Cult Temperat Block Perc24h

Bt 28 1 93.33

Bt 28 2 91.67

Bt 28 3 100.00

Bt 28 4 65.22

Bt 28 5 95.65

Is 28 1 100.00

Is 28 2 96.77

Is 28 3 92.00

Is 28 4 96.67

Is 28 5 86.21
