# Supplementary material for: Feeding and Dispersal Behavior of the Cotton Leafworm, Alabama argillacea (Hübner) (Lepidoptera: Noctuidae), on Bt and Non-Bt Cotton: Implications for Evolution and Resistance Management
Source: PLoS One. 2014 Nov 4;9(11):e111588. doi: 10.1371/journal.pone.0111588 (PMC4219722; doi:10.1371/journal.pone.0111588)
Supplement: Data Set S3 — Data set for neonate larvae recovered dead after 24 h. (DOCX) [file pone.0111588.s003.docx]

**Data Set S3.** Data set for neonate larvae recovered dead after 24 h.

Cult = Cultivar

Temperat = Temperature

Block

M24h = Mortality after 24 h

Bt = Bt cotton plant

IS = non-BT cotton plant

Cult Temperat Block M24h

Bt 28 1 46.43

Bt 28 2 60.00

Bt 28 3 23.33

Bt 28 4 23.33

Bt 28 5 23.33

Is 28 1 0.00

Is 28 2 0.00

Is 28 3 16.67

Is 28 4 11.76

Is 28 5 3.33
