# Supplementary material for: Feeding and Dispersal Behavior of the Cotton Leafworm, Alabama argillacea (Hübner) (Lepidoptera: Noctuidae), on Bt and Non-Bt Cotton: Implications for Evolution and Resistance Management
Source: PLoS One. 2014 Nov 4;9(11):e111588. doi: 10.1371/journal.pone.0111588 (PMC4219722; doi:10.1371/journal.pone.0111588)
Supplement: Data Set S4 — Data set for neonate larvae that had fed found on plant and on bag (%). (DOCX) [file pone.0111588.s004.docx]

**Data Set S4**. Data set for neonate larvae that had fed found on plant and on bag (%).

*Forat* = outside (transformated)

*Plantat* = on plant (transformated)

data proportion;

input Temperature$ Cultivar$ Block$ Time$ Outside (bag) Forat Plantat Plantat;

datalines;

28 OBt 1 O6 0.0000 0.0000 1.0000 1.0000

28 OBt 2 O6 0.0000 0.0000 1.0000 1.0000

28 OBt 3 O6 0.0000 0.0000 1.0000 1.0000

28 OBt 4 O6 0.0000 0.0000 1.0000 1.0000

28 OBt 1 12 0.0000 0.0000 1.0000 1.0000

28 OBt 2 12 0.0000 0.0000 0.7200 0.8485

28 OBt 3 12 0.0000 0.0000 1.0000 1.0000

28 OBt 4 12 0.0000 0.0000 1.0000 1.0000

28 OBt 1 18 0.5000 0.7071 1.0000 1.0000

28 OBt 2 18 0.5000 0.7071 1.0000 1.0000

28 OBt 3 18 0.5000 0.7071 1.0000 1.0000

28 OBt 4 18 0.5000 0.7071 1.0000 1.0000

28 OBt 1 24 0.5000 0.7071 1.0000 1.0000

28 OBt 2 24 0.5000 0.7071 1.0000 1.0000

28 OBt 3 24 0.5000 0.7071 1.0000 1.0000

28 OBt 4 24 0.5000 0.7071 1.0000 1.0000

28 NBt 1 O6 0.0000 0.0000 1.0000 1.0000

28 NBt 2 O6 0.5000 0.7071 1.0000 1.0000

28 NBt 3 O6 0.5000 0.7071 1.0000 1.0000

28 NBt 4 O6 0.5000 0.7071 1.0000 1.0000

28 NBt 1 12 0.5000 0.7071 1.0000 1.0000

28 NBt 2 12 0.2500 0.5000 1.0000 1.0000

28 NBt 3 12 0.7500 0.8660 1.0000 1.0000

28 NBt 4 12 0.5000 0.7071 1.0000 1.0000

28 NBt 1 18 0.5000 0.7071 1.0000 1.0000

28 NBt 2 18 0.2000 0.4472 1.0000 1.0000

28 NBt 3 18 0.8000 0.8944 0.6669 0.8166

28 NBt 4 18 0.5000 0.7071 1.0000 1.0000

28 NBt 1 24 0.0000 0.0000 1.0000 1.0000

28 NBt 2 24 0.0000 0.0000 1.0000 1.0000

28 NBt 3 24 0.0000 0.0000 1.0000 1.0000

28 NBt 4 24 0.0000 0.0000 1.0000 1.0000
