# Supplementary material for: Feeding and Dispersal Behavior of the Cotton Leafworm, Alabama argillacea (Hübner) (Lepidoptera: Noctuidae), on Bt and Non-Bt Cotton: Implications for Evolution and Resistance Management
Source: PLoS One. 2014 Nov 4;9(11):e111588. doi: 10.1371/journal.pone.0111588 (PMC4219722; doi:10.1371/journal.pone.0111588)
Supplement: Data Set S5 — Data set for plant tissue in gut of neonate larvae recovered from cotton plant. (DOCX) [file pone.0111588.s005.docx]

**Data Set S5.** Data set for plant tissue in gut of neonate larvae recovered from cotton plant.

Constrans = food in gut

Consobs = food in gut (transformated)

data consnaplant;]

input Tra$ Te$ Ti$ B Constrans Consobs;

datalines;

OBt 28 O6 1 1.6092 2.5897

OBt 28 O6 2 1.9950 3.9800

OBt 28 O6 3 1.5731 2.4745

OBt 28 O6 4 2.4117 .8163

OBt 28 12 1 2.6187 6.8576

OBt 28 12 2 2.3411 5.4807

OBt 28 12 3 2.5646 6.5770

OBt 28 12 4 1.9913 3.9653

OBt 28 18 1 2.3142 5.3557

OBt 28 18 2 2.3905 5.7146

OBt 28 18 3 2.3786 5.6579

OBt 28 18 4 3.3307 11.093

OBt 28 24 1 2.9444 8.6693

OBt 28 24 2 3.0410 9.2475

OBt 28 24 3 2.7260 7.4309

OBt 28 24 4 2.7260 7.4309

NBt 28 O6 1 2.9806 8.8837

NBt 28 O6 2 2.3869 5.6975

NBt 28 O6 3 3.3667 11.334

NBt 28 O6 4 3.9343 15.479

NBt 28 12 1 3.3307 11.093

NBt 28 12 2 3.7646 14.172

NBt 28 12 3 3.1403 9.8613

NBt 28 12 4 3.3346 11.119

NBt 28 18 1 3.9672 15.738

NBt 28 18 2 4.6626 21.740

NBt 28 18 3 3.9057 15.254

NBt 28 18 4 3.7366 13.962

NBt 28 24 1 5.0746 25.752

NBt 28 24 2 4.4649 19.935

NBt 28 24 3 4.9407 24.410

NBt 28 24 4 5.9213 35.061
