# Supplementary material for: Feeding and Dispersal Behavior of the Cotton Leafworm, Alabama argillacea (Hübner) (Lepidoptera: Noctuidae), on Bt and Non-Bt Cotton: Implications for Evolution and Resistance Management
Source: PLoS One. 2014 Nov 4;9(11):e111588. doi: 10.1371/journal.pone.0111588 (PMC4219722; doi:10.1371/journal.pone.0111588)
Supplement: Data Set S6 — Data set for plant tissue in gut of neonate larvae found on bag (μm2). (DOCX) [file pone.0111588.s006.docx]

**Data Set S6.** Data set for plant tissue in gut of neonate larvae found on bag (μm^2^).

Constrans = food in gut (transformated)

data Consoutside;

input Temperature$ Block$ Time$ Cultivar$ food in gut Constrans;

datalines;

28 1 O6 OBt 0.00 0.00

28 2 O6 OBt 0.00 0.00

28 3 O6 OBt 0.00 0.00

28 4 O6 OBt 0.00 0.00

28 1 12 OBt 0.00 0.00

28 2 12 OBt 0.00 0.00

28 3 12 OBt 0.00 0.00

28 4 12 OBt 0.00 0.00

28 1 18 OBt 4.41 2.10

28 2 18 OBt 2.69 1.64

28 3 18 OBt 3.55 1.88

28 4 18 OBt 3.98 1.99

28 1 24 OBt 3.61 1.90

28 2 24 OBt 7.65 2.77

28 3 24 OBt 8.92 2.99

28 4 24 OBt 2.06 1.43

28 1 O6 NBt 6.24 2.50

28 2 O6 NBt 4.00 2.00

28 3 O6 NBt 1.76 1.33

28 4 O6 NBt 4.00 2.00

28 1 12 NBt 7.94 2.82

28 2 12 NBt 1.08 1.04

28 3 12 NBt 4.51 2.12

28 4 12 NBt 4.50 2.12

28 1 18 NBt 18.2 4.27

28 2 18 NBt 10.1 3.18

28 3 18 NBt 14.1 3.77

28 4 18 NBt 14.1 3.77

28 1 24 NBt 0.00 0.00

28 2 24 NBt 0.00 0.00

28 3 24 NBt 0.00 0.00

28 4 24 NBt 0.00 0.00
